# Long-term exposure to commercially available sunscreens containing nanoparticles of TiO_2_ and ZnO revealed no biological impact in a hairless mouse model

**DOI:** 10.1186/s12989-016-0154-4

**Published:** 2016-08-17

**Authors:** Megan J. Osmond-McLeod, Yalchin Oytam, Anthony Rowe, Fariborz Sobhanmanesh, Gavin Greenoak, Jason Kirby, Elizabeth F. McInnes, Maxine J. McCall

**Affiliations:** 1 CSIRO Advanced Materials TCP (Nanosafety), Manufacturing Flagship, North Ryde, NSW 2113 Australia; 2 CSIRO, Food & Nutrition Flagship, North Ryde, NSW 2113 Australia; 3 CSIRO Land & Water Flagship, Urrbrae, SA 5064 Australia; 4 Australian Photobiology Testing Facility, Forest Lodge, NSW 2037 Australia; 5 Gribbles Veterinary Pathology Australia, Glenside, SA 5065 Australia; 6 PO Box 52, North Ryde, NSW 1670 Australia

**Keywords:** Long-term, Sunscreen, Nanoparticles, Organic UV filter, Ultraviolet radiation

## Abstract

**Background:**

The application of sunscreen is a critical component of a sun-safe strategy, however the possibility of unexpected, adverse outcomes resulting from long-term use of sunscreens containing nanoparticles of titanium dioxide (TiO_2_) and zinc oxide (ZnO) has not yet been examined. Here, immune-competent hairless mice were exposed over a 36-week period to weekly topical applications of sunscreens containing nanoparticles of ZnO or TiO_2_, or no metal oxide nanoparticles, with or without subsequent exposure to ultraviolet radiation (UVR). Control groups received no sunscreen applications, with or without UVR.

**Results:**

Mice exposed to UVR in the absence of sunscreen developed statistically significant incidences of histologically-diagnosed malignant and benign skin neoplasms, whereas no statistically significant adverse biological outcomes were found in mice treated with the sunscreens containing ZnO or TiO_2_ nanoparticles. Elevated levels of Ti were detected in the livers of mice treated with sunscreen containing TiO_2_ nanoparticles compared to untreated control, but total Zn concentrations did not significantly alter in any major organs except for the skin of mice treated with ZnO sunscreen. Exposure to UVR did not have a significant impact on examined tissue concentrations of Zn or Ti. Few to no transcriptional changes were found in ZnO or TiO_2_-treated groups, but mice treated with the sunscreen containing only organic filters showed substantial gene disregulation.

**Conclusions:**

Taken together with previous work, this long-term study provided no basis to avoid the use of sunscreens containing metal oxide nanoparticles.

## Background

Whilst some exposure of naked skin to sunlight is beneficial for human health [1], the demonstrated consequences of prolonged exposure of unprotected skin to ultraviolet radiation (UVR) include inflammation, premature photo-ageing, DNA damage, photocarcinogenesis, and immune suppression [2, 3]. The regular application of sunscreen has been shown to decrease the risk of developing squamous cell carcinomas in humans, as well as correlated with decreased risk of developing melanomas and basal cell carcinomas [4]. Therefore, when long periods outdoors are anticipated, the regular application of sunscreens is encouraged (e.g. [5–7]).

The active ingredients in topically-applied sunscreens generally fall into two categories: organic or inorganic. Organic UVR filters typically contain aromatic ring structures that absorb radiation in the UV waveband and may bear functional groups that act as electron donors and receptors following exposure to UVR. In contrast, inorganic metal oxide particles, typically titanium dioxide (TiO_2_) or zinc oxide (ZnO), form a physical barrier between the skin and UVR. Both organic and inorganic filters have been used in sunscreens for decades, but the latter were typically confined to small areas of high exposure, such as the nose, due to their opaque appearance on skin resulting from the use of pigment-sized particles. Nano-sized (defined here as having at least one dimension <100 nm [8]) particles of TiO_2_ and ZnO have been used in sunscreens since the 1980s, becoming more widespread from the 1990s [9]. Their development has resulted in modern formulations that are highly effective UVR filters yet are also transparent and light-textured on the skin [9], superseding the thick, white creams of yesteryear. Whilst ZnO is protective against both UVB and UVA, TiO_2_ is typically partnered with organic filters to achieve broad spectrum protection.

The European Union (EU) Commission’s Working Group on Cosmetics recently concluded, on the basis of comprehensive safety dossiers, that the inclusion of nano-sized ZnO [10] and TiO_2_ [11] up to 25 % in dermally-applied cosmetic products posed no risk for adverse health effects in humans. Similarly, Australia’s Therapeutic Goods Administration (TGA) has twice concluded that there is no evidence to show that TiO_2_ and ZnO nanoparticles are not safe for human use in sunscreens [12, 13], and a comprehensive review commissioned by the Danish Environmental Protection concluded that topically applied nanoparticles are unlikely to undergo dermal penetration under most conditions, although some conditions may enhance penetration to a very small degree [14]. The majority of scientific reviews in peer-reviewed journals have reached similar conclusions. Thus, we can suggest that a scientific consensus is being reached that nanoparticles in sunscreens are safe for use in humans. Nevertheless, some uncertainty remains with respect to possible biological impacts arising from long-term use of these sunscreens [11]. This, combined with the novelty of nanotechnology, has led to speculation within some sections of the community that nanoparticle-containing sunscreens may be unsafe in the long-term (e.g. [15]) despite clear evidence that the avoidance of sunscreens altogether can leave the skin exposed to the known damaging effects of UVR. This study, which specifically compares the biological outcomes of long-term intermittent UVR exposure, with or without the protection of topically-applied sunscreens containing either metal oxide nanoparticles and/or organic filters, addresses some of these concerns.

An immune-competent hairless mouse model was chosen to provide a highly sensitive model of skin penetration relative to humans [16], whilst retaining a comparable response profile to UVR [17–22]. Three types of commercially-available sunscreens were applied to the backs of mice over the course of 36 wks, with and without exposure to UVR (Series 1). One sunscreen contained only ZnO nanoparticles as the active ingredient, the second contained a mixture of TiO_2_ nanoparticles and organic active ingredients [octylmethoxycinnamate (OCM); butyl methoxydibenzoylmethane (B-MDM)], and the third contained only organic active ingredients (OCM, 99 mg/mL; B-MDM, 19.8 mg/mL; (4-methylbenzylidene camphor (4-MBC), 39.6 mg/mL; octocrylene, 9.9 mg/mL). A follow-up study (Series 2) assessed only control groups (no sunscreen ± UVR) compared to ZnO treatment groups (± UVR). The mice wore Elizabethan collars during treatment periods to limit ingestion of sunscreen by licking.

## Results

Treatment groups in Series 1 and 2 are summarised in Table 1.

**Table 1:** Treatment groups in Series 1 and Series 2

	Group ID	Treatment
Series 1	Control-UVR	No sunscreen, no UVR
	Control + UVR	No sunscreen + 29 kJ/m^2^ UVR
	ZnO-UVR	ZnO sunscreen, no UVR
	ZnO + UVR	ZnO sunscreen + 29 kJ/m^2^ UVR
	TiO_2_-UVR	TiO_2_ sunscreen, no UVR
	TiO_2_ + UVR	TiO_2_ sunscreen + 29 kJ/m^2^ UVR
	Organic-UVR	Organic sunscreen, no UVR
	Organic + UVR	Organic sunscreen + 29 kJ/m^2^ UVR
Series 2	2-Control-UVR	No sunscreen, no UVR
	2-Control + UVR	No sunscreen + 27 kJ/m^2^ UVR
	2-ZnO-UVR	ZnO sunscreen, no UVR
	2-ZnO + UVR	ZnO sunscreen + 27 kJ/m^2^ UVR

### Characterisation of nanoparticles extracted from sunscreens

**Fig. 1:**
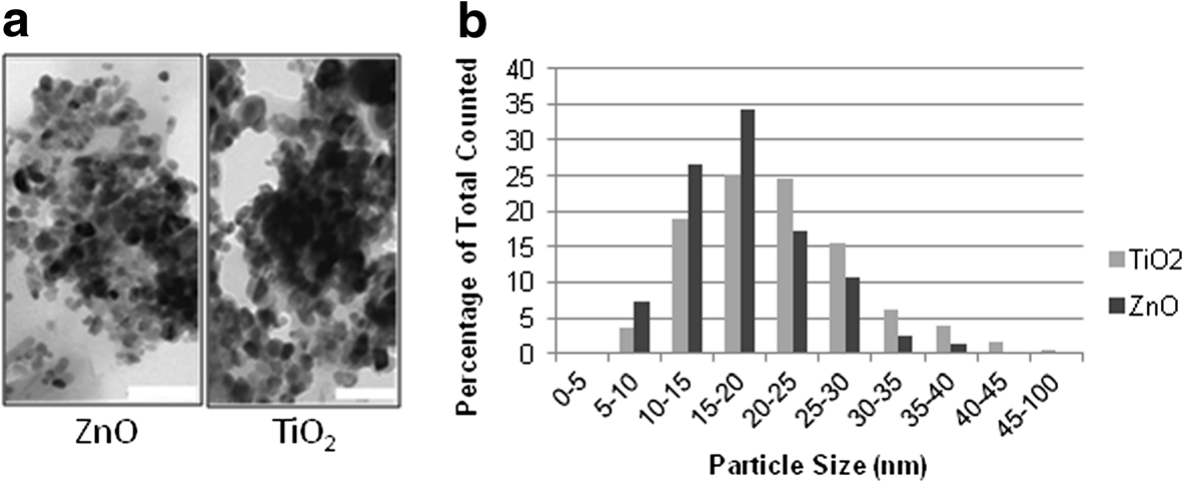
**a** TEM images of ZnO and TiO_2_ particles extracted from their respective sunscreens. Scale bar = 100 nm; **b** Histogram showing the distribution of particle diameters. 233 and 257 measurements were made for ZnO and TiO_2_ particles, respectively

The morphologies and size distributions of nanoparticles extracted from the ZnO and TiO_2_ sunscreens are shown in Fig. 1. Both ZnO and TiO_2_ particles were typically spheroidal in appearance, and had average diameters of 18.2 ± 0.4 nm (*n* = 233), and 21.5 ± 0.6 nm (*n* = 257), respectively. A two-tailed *t*-test showed that the ZnO particles were significantly smaller than the TiO_2_ (*p* < 0.0001). However, as the shapes and sizes of the ZnO and TiO_2_ nanoparticles were comparable, any difference in their effects is likely to be due to their chemical composition and related properties.

### Macroscopic and histopathological outcomes in mice

On completion of both the Series 1 and Series 2 experiments, mice receiving treatments of UVR with no sunscreen had developed significantly thicker skin in the dorsal region (Control + UVR): 2.5 ± 0.4 mm, *n* = 5; 2-Control + UVR: 2.1 ± 0.2 mm, *n* = 8) compared to untreated mice (Control-UVR: 0.75 ± 0.02 mm, *n* = 10; 2-Control-UVR: 0.83 ± 0.01 mm, *n* = 10) (*p* < 0.0001). Other treatment groups did not show a similar skin thickening, with means ranging from 0.74 ± 0.01 (ZnO-UVR, *n* = 10) to 0.77 ± 0.04 (TiO_2_ + UVR, *n* = 10) in Series 1, and 0.82 ± 0.02 (2-ZnO-UVR, *n* = 10) to 0.86 ± 0.02 (2-ZnO + UVR, *n* = 9) in Series 2. Non-malignant skin neoplasms (sessile-based papillomas, macules, and pedunculated papillomas) were identified macroscopically for Series 1 on the dorsa of 4 of the 10 mice from Control + UVR, but were not assessed in Series 2. Mice showed no statistically significant difference in average body or organ weights, and no other macroscopic outcomes were observed.

Of the total of 120 mice used across Series 1 and 2, 105 maintained good health throughout the experimental periods and reached the pre-determined experimental endpoints of 36 weeks for both Series. Of these 105 mice, 86 were classified as normal and 19 were diagnosed with sub-clinical problems in a post-mortem analysis performed by a veterinary histopathologist employed on a commercial basis. Details of histopathological findings are given in Table 2. A total of 15 of the 120 mice used across Series 1 and Series 2 suffered unscheduled adverse events and were either euthanized for ethical reasons (12 mice) or were found dead in the cage (3 mice), and thus did not reach the pre-determined experimental endpoints.

**Table 2:** Histological findings for all treatment groups from Series 1 and Series 2

	Group ID	Sunscreen	UVR kJ/m^2^	Number of mice per group	Number of mice that reached endpoint without AE	Total mice with AE	Mice with AE related to malignant or pre-malignant findings (histologically diagnosed)	Mice with AE related to non-malignant findings (histologically diagnosed)	Mice with AE, no necroscopy	Total mice at predetermined endpoint with malignant findings (histologically diagnosed)	Total mice at predetermined endpoint with non-malignant findings (histologically diagnosed)	Total mice at predetermined endpoint with no malignant or non-malignant findings
Series 1	Control-UVR	None	0	10	10	0	0	0	0	0	0	10
	Control + UVR	None	29	10	6	4	4 (1 X CFS; 1 X CFS + SCC; 1 X LL; 1 X SCP)	0	0	1 (1 X SCP)	4^a^	1
	ZnO-UVR	ZnO	0	10	10	0	0	0	0	0	1 (1 X PA)	9
	ZnO + UVR	ZnO	29	10	5	5	1 (1 X LL)	2 (1 X N; 1 X HC)	2	1 (1 X CFS)	0	4
	TiO_2_-UVR	TiO_2_	0	10	9	1	0	1 (1 X P)	0	0	0	9
	TiO_2_ + UVR	TiO_2_	29	10	10	0	0	0	0	2 (2 X BAC)	1 (1 X PA)	7
	Organic-UVR	Organic	0	10	9	1	1 (1 X LL)	0	0	0	0	9
	Organic + UVR	Organic	29	10	9	1	0	0	1	1 (1 X LL)	0	8
Series 2	2-Control-UVR	None	0	10	10	0	0	0	0	0	0	10
	2-Control + UVR	None	27	10	9	1	1 (1 X LL)	0	0	7 (1 X BAC; 4 X SCC; 1 X LL; 1 X CFS & SCP)	0	2
	2-ZnO-UVR	ZnO	0	10	9	1	1 (1 X LL)	0	0	1 (1 X OS)	0	8
	2-ZnO + UVR	ZnO	27	10	9	1	0	1 (1 X E)	0	0	0	9

The predetermined endpoint for both groups was 36 weeks

*CODE*: *AE* adverse event, *BAC* bronchioalveolar carcinoma, *CFS* cutaneous fibrosarcoma, *E* endometritis, *HC* haemolytic crisis, *LL* lymphocytic lymphoma, *N* nephropathy, *OS* osteosarcoma, *P* peritonitis, *PA* pulmonary adenoma, *SCC* squamous cell carcinoma, *SCP* squamous cell papilloma

^a^No malignant or non-malignant outcomes, but moderate to severe dermal hyperplasia and inflammation were histologically diagnosed in all four mice

**Fig. 2:**
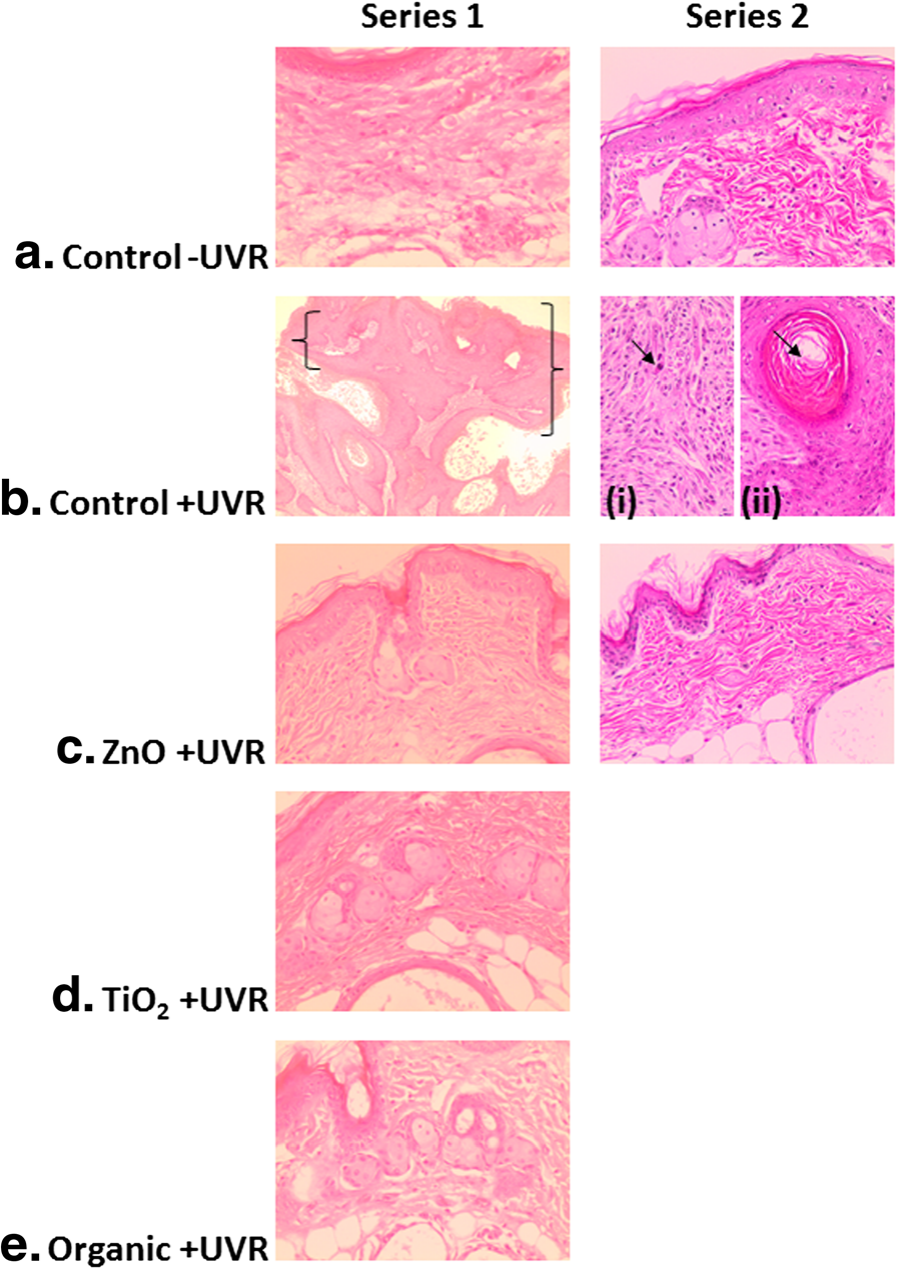
Images of representative histological skin sections from mice from Series 1 and 2 receiving: **a** no treatment; **b** weekly exposure to UVR with no sunscreen; **c** weekly exposure to UVR with pre-applications of sunscreen containing nanoparticles of ZnO as the only UV active ingredient; **d** weekly exposure to UVR with pre-application of sunscreen containing nanoparticles of TiO2 and organic UV active ingredients; **e** weekly exposure to UVR with pre-application of sunscreen containing only organic UV active ingredients. Images on the left were taken from mice in Series 1, and on the right in Series 2. The only treatment-specific effects were in the mice receiving UVR treatments with no sunscreen (Control + UVR), here represented by a squamous cell papilloma (SCP) in Series 1 (encompassed by a black bracket), and in Series 2 (i) a cutaneous fibrosarcoma (CFS) (*black arrow* indicates mitotic figure in fibrosarcoma) and (ii) squamous cell carcinoma (SCC) (*black arrow* indicates “keratin pearl” in SCC). The skin from all other treatment groups showed unremarkable epidermis and dermis

Apart from the expected UVR-induced dermal neoplasms observed for mice in the UV-irradiated groups receiving no sunscreen (Control + UVR, 2-Control + UVR) (Fig. 2), post-mortem examinations found no statistically-significant common cause to any of the histologically-diagnosed pathologies in mice that reached the experimental endpoint or suffered unscheduled adverse events across Series 1 and Series 2.

### Levels of tissue Zn

The levels of Zn were measured by ICP-MS in the skin, brain, liver, spleen, kidney, and lung tissues from control and ZnO-treated groups in Series 1, and are shown in Table 3. Mice receiving weekly topical applications of sunscreen containing ZnO nanoparticles showed elevated concentrations of Zn in the skin (ZnO-UVR: 82 ± 9 μg/g; ZnO + UVR: 73 ± 10 μg/g) compared to mice receiving no sunscreen (Control-UVR: 14.5 ± 0.7 μg/g; Control + UVR: 13 ± 2 μg/g). No difference was found between ZnO-UVR and ZnO + UVR. Elevated concentrations of Zn were not found in any of the other tissues examined.

**Table 3:** Mean concentrations of Zn in tissues (wet mass) from untreated mice, and treated mice receiving UVR only, or ZnO sunscreen (± UVR) in Series 1

	Group ID	Sunscreen	UVR (kJ/m^2^)	μg/kg	Mean μg/g ± SEM					
					Skin	Brain	Liver	Spleen	Kidney	Lung
Series 1	Control-UVR	None	None	Zn	14.5 ± 0.7	12.2 ± 0.8	19 ± 1	17.4 ± 0.5	14.8 ± 0.7	16.9 ± 0.5
	Control + UVR	None	29	Zn	13 ± 2	15 ± 3	20 ± 1	17.6 ± 0.6	15.7 ± 0.8	16.6 ± 0.6
	ZnO-UVR	ZnO	None	Zn	82 ± 9^a^	12.1 ± 0.6	21 ± 1	18.2 ± 0.1	16.6 ± 0.6	16.1 ± 0.4
	ZnO + UVR	ZnO	29	Zn	73 ± 10^a^	13 ± 1	21 ± 1	17.3 ± 0.5	17.3 ± 0.7	16.4 ± 0.4

The practical quantification limit of Zn was 0.4 μg/kg; *n* = 5 for all tissues in all groups except for Control + UVR Skin, for which *n* = 4

*SEM* Standard Error of the Mean

^a^Significantly different from untreated mice (Control-UVR)

### Levels of Liver Ti

**Fig. 3:**
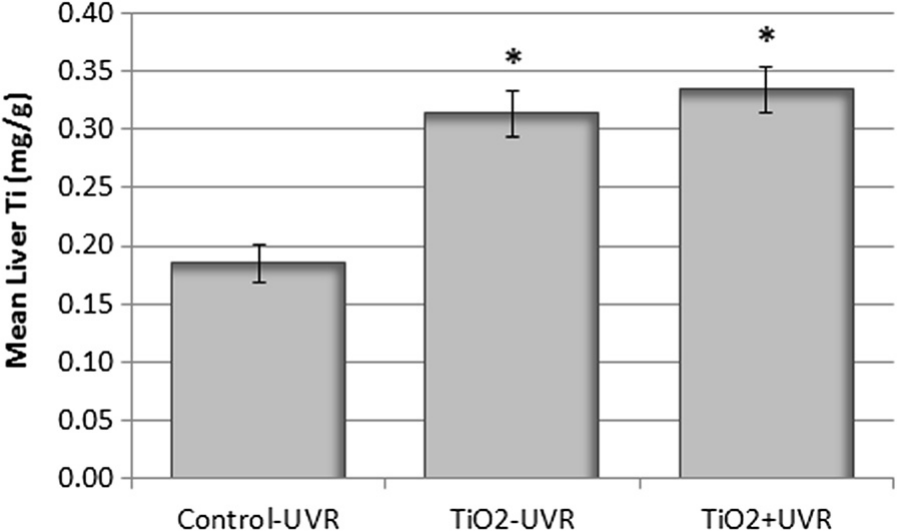
Mean concentrations of Ti in livers (dry mass) of untreated mice, and mice receiving TiO_2_ sunscreen (± UVR) in Series 1. *Significantly different from untreated mice (Control-UVR) (*p* < 0.001)

Total Ti concentrations were measured by ICP-MS in liver tissues from Control-UVR, TiO_2_-UVR, and TiO_2_ + UVR, and are shown in Fig. 3. Mice exposed to topical applications of sunscreen containing TiO_2_ nanoparticles once per week over 36 weeks (TiO_2_-UVR and TiO_2_ + UVR) showed significantly elevated levels of Ti in liver tissue (0.31 ± 0.02 μg/g and 0.33 ± 0.02 μg/g, respectively) compared to untreated mice (Control-UVR: 0.19 ± 0.2 μg/g). No difference was found between TiO_2_-UVR and TiO_2_ + UVR. The source of background Ti (i.e. in untreated mice) is likely to have been food, which was analysed and found to contain 0.3 mg/kg Ti.

### Whole-genome gene-expression profiling in livers

No statistically-significant differentially-expressed transcripts were identified in ZnO-UVR, ZnO + UVR, or TiO_2_-UVR compared to Control-UVR, and so these groups were excluded from further transcript analysis. In contrast, statistically significant alterations in transcript levels were identified in mice from Control + UVR, TiO_2_ + UVR, Organic-UVR, and Organic + UVR for a subset of genes compared to untreated mice, and thus further analyses were performed for these groups using IPA software to identify perturbed transcriptional pathways.

**Fig. 4:**
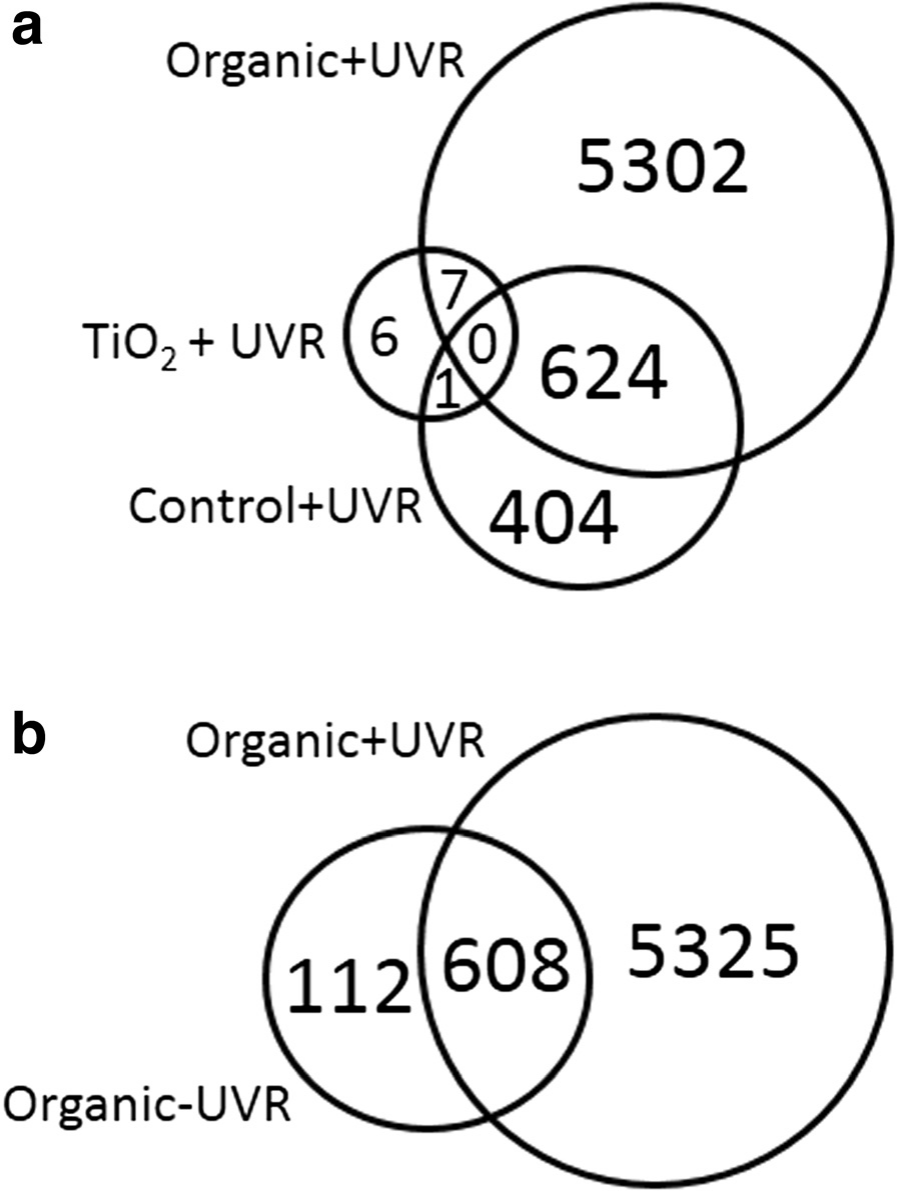
**a** VENN diagram showing the number of unique or shared transcripts within and between mice exposed to UVR, with (TiO_2_ + UVR, Organic + UVR) or without (Control + UVR) prior application of sunscreen. Numbers indicate differentially-expressed transcripts that were either unique to one treatment (unshared VENN), or differentially-expressed in more than one treatment (intersecting VENN); **b** VENN diagram showing the number of unique or shared transcripts within and between mice receiving topical applications of the organic sunscreen, with (Organic + UVR) or without (Organic-UVR) subsequent UVR exposure

As depicted in Fig. 4, mice receiving the combination of organic sunscreen and UVR (Organic + UVR) showed by far the greatest disruption to the transcriptome, with a total of 5933 genes differentially regulated compared to untreated mice (Control-UVR). In the absence of either the organic sunscreen (Control + UVR) or the UVR (Organic-UVR), mice exhibited far less disturbance to their transcriptomic activity, with totals of 1029 and 720 genes differentially regulated, respectively. In contrast, mice treated with both the TiO_2_ sunscreen and UVR (TiO_2_ + UVR) showed only very low levels of differential regulation compared to untreated mice (Control-UVR) (14 genes).

Information on all differentially-expressed genes from all treatment groups relative to no treatment is available under GEO accession number GSE84818. The top 5 Canonical Transcriptional Pathways showing the most substantial disruption in treatment groups are listed in Table 4, although it should be noted that the identification of canonical pathways by IPA and similar programs that are based on gene and pathway citations in research literature can be subject to bias towards areas of greater research effort and literature abundance. Three of the five most substantially perturbed by UVR treatment in the absence of sunscreen (Control + UVR) were associated with the response to DNA damage (Role of BRCA1 in DNA Damage Response; ATM signalling; DNA Double-Strand Break Repair by Non-Homologous End Joining), and a fourth (NF-kB Signaling) was a rapid response pathway to cellular damage. The fifth, Cholecystokinin/Gastrin-Mediated Signalling, is a molecular messaging pathway implicated in a variety of biological functions that, amongst others, include cell proliferation and neoplastic transformation. In contrast, three of the Top 5 Canonical Pathways that were disrupted in mice receiving topical applications of TiO_2_ sunscreen prior to UVR (TiO_2_ + UVR) were associated with metabolic functions (Tetrapyrrole Biosynthesis II; Heme Biosynthesis II; Mevalonate Pathway I). The Circadian Rhythm Signalling and Breast Cancer Regulation by Stathmin1 Canonical Pathways also showed altered regulation in this group. Mice receiving topical treatments of the organic sunscreen with (Organic + UVR) or without (Organic-UVR) subsequent UVR exposure shared two of their Top 5 Canonical Pathways, with the Estrogen Receptor Signalling and Remodeling of Epithelial Adherens Junctions Pathways showing substantially altered regulation in both. However, in the absence of UVR (Organic-UVR), two of the remaining three Top 5 Pathways were associated with metabolic functions (All-trans-decaprenyl Diphosphate Biosynthesis; Phosphatidylethanolamine Biosynthesis II), whereas in the presence of UVR (Organic + UVR) the remaining three pathways in the Top 5 were indicative of altered protein regulation (Protein Ubiquitination Pathway; tRNA Charging; EIF2 Signaling). It should be noted, however, that whilst the different treatment regimes resulted in variation amongst the Top 5 Canonical Pathways, these pathways were often also disrupted by the other treatments, but not as strongly.

**Table 4:** Top 5 canonical transcriptional pathways perturbed in treated, relative to untreated, mice from Series 1

Group ID	Sunscreen	UVR (kJ/m^2^)	Top 5 canonical pathways
Control + UVR	None	29	Role of BRCA1 in DNA Damage Response; Cholecystokinin/Gastrin-Mediated Signalling; ATM Signalling; NF-kB Signalling; DNA Double-Strand Break Repair by Non-Homologous End Joining
ZnO-UVR	ZnO	0	No statistically significant differentially expressed genes identified
ZnO + UVR	ZnO	29	No statistically significant differentially expressed genes identified
TiO_2_-UVR	TiO_2_	0	No statistically significant differentially expressed genes identified
TiO_2_ + UVR	TiO_2_	29	Circadian Rhythm Signalling; Tetrapyrrole Biosynthesis II; Heme Biosynthesis II; Breast Cancer Regulation by Stathmin1; Mevalonate Pathway I
Organic-UVR	Organic	0	Estrogen Receptor Signalling; UVA-induced MAPK Signalling; All-trans-decaprenyl Diphosphate Biosynthesis; Remodelling of Epithelial Adherens Junctions; Phosphatidylethanolamine Biosynthesis II
Organic + UVR	Organic	29	Protein Ubiquitination Pathway; tRNA Charging; Estrogen Receptor Signalling; Remodelling of Epithelial Adherens Junctions; EIF2 Signalling

## Discussion

This study sought to address the question of whether long-term use of sunscreens containing metal oxide nanoparticles could lead to adverse health effects compared to a sunscreen containing only organic UVR filters, or no sunscreen at all. As sunscreen is typically used to protect the skin from the sun, it was important to include UVR as a variable, particularly in light of the photocatalytic potential of ZnO and TiO_2_ nanoparticles and the potential for temporary disruption of the skin barrier function following UVR exposure. Two 36-week experiments were conducted in which immune-competent hairless mice received weekly topical applications of commercially-available sunscreens, or no sunscreen, followed by UVR exposure or no UVR.

The major findings of the study are aligned with general sun-safe messages that advocate the application of sunscreen to skin when exposed to UVR in the absence of protective clothing; i.e. sunscreen protects the skin against UVR-mediated damage. These results also counter recent speculations within some sections of the community that sunscreens containing metal oxide nanoparticles may be more dangerous than no sunscreen at all [23]. We found that the topical use of sunscreens protected hairless mice from histologically-diagnosed UVR-induced malignant and non-malignant skin neoplasms, and dermal inflammation, irrespective of whether their active ingredients included metal oxide nanoparticles and/or organic filters. This is consistent with previously published work showing that sunscreen use can decrease incidences of solar actinic keratoses [24], melanoma [25], and squamous and basal cell carcinomas [26] in humans. We found no other statistically-significant histological outcomes with respect to the long-term use of ZnO or TiO_2_ nanoparticles or organic filters in sunscreens. These results thus support the recent position of the Scientific Committee on Consumer Safety that TiO_2_ and ZnO nanoparticles in sunscreens are not considered to be harmful to humans after topical application [11, 27].

Dermal penetration by nanoparticles in sunscreen was not the focus of this study and was not controlled as we cannot exclude the possibility of chronic low-level ingestion of residual sunscreen after washing each week. Bearing this in mind, the presence of UVR here did not significantly alter the concentrations of Zn in brain, heart, liver, kidney, or spleen tissue, or Ti in liver, compared to the concentrations in mice receiving sunscreen applications without UVR. This suggests that, with long-term sunscreen use, UVR does not substantially alter the dermal penetrability of metal oxide nanoparticles when sunscreens are applied prior to UVR exposure.

Internal tissue concentrations of Zn were not elevated in mice treated with the ZnO sunscreen, whereas the concentration of Ti was slightly elevated in the livers of mice treated with the TiO_2_ sunscreen. Whilst it is not possible to exclude dermal penetration, a far more likely route of entry was via ingestion of either particles from licking each other during treatment periods (although this was not observed), or from licking residual particles left in hair follicles and skin furrows after washing during routine grooming (i.e. after the Elizabethan collars had been removed each week). The difference between the absence of Zn accumulation and observed low-level accumulation of Ti in the livers may reflect the differing solubilities of the more soluble ZnO compared to the much less soluble TiO_2_ nanoparticles, highlighting that the fate of nanoparticles following internalisation into organs and cells may differ according to their physical and chemical characteristics. Alternatively, if the same (low) levels of Zn and TiO_2_ were internalised dermally or via ingestion, it would be easier to detect a measureable change in concentration for Ti than Zn, simply due to the very different background or endogenous concentrations which are much higher for Zn, where Zn is an endogenous metal cofactor with normal homestatic mechanisms, whereas Ti is a contaminant needing hepatic decontamination and egestion. Indeed, this was the reason for using the stable isotope method in previous work where one of the aims was to assess dermal penetration of Zn from ZnO sunscreen in humans and mice [28]. Interestingly, however, Cho et al. [29] reported higher absorption, organ distribution, and urine excretion of Zn from orally-administered ZnO nanoparticles in mice, compared to TiO_2_, which was largely expelled via the faeces. This difference may reflect the differing route of administration and doses used. We did not assess tissue concentrations of the organic filters here, but work elsewhere has suggested that they may undergo time- and dose-dependent systemic absorption in humans [30, 31].

As might be anticipated, the transcriptional pathways most substantially disrupted by treatment with UVR without protection from sunscreen were associated with responses to inflammation and DNA damage. In contrast, for the treatments with the organic sunscreen with or without UVR, the most significantly altered pathways were associated with hormone signalling, cell signalling, and protein synthesis. UVR does not appear to be a factor in increasing absorption of organic UVR filters [31], and therefore the dramatic increase in transcriptional disruption in mice treated with the organic sunscreen with UVR compared to without UVR is interesting. This could be due to either compounds produced in organic sunscreen during irradiation, or production of compounds by the skin when in contact with organic sunscreen during irradiation. To distinguish between these possibilities, future work could include a group where the organic sunscreen is treated with UVR before being placed on the skin of mice which are not subsequently exposed to UVR.

Interestingly, long-term exposure to the TiO_2_ sunscreen induced very few changes to the transcriptional profile, despite having two organic UV filters in common with the organic-only sunscreen. This may indicate that the transcriptional changes observed in the organic sunscreen-treated mice may have been largely mediated by the two additional filters that were present only in the organic sunscreen. Of those, octocrylene is not currently known to have endocrine-disrupting activity, but 4-MBC has been demonstrated to induce dose-dependent estrogenic activity in vivo in rodent models when applied orally or topically [32, 33]. However, it is important to note that in a *human* study where young adult males or post-menopausal women were treated with 4-MBC daily over 2 weeks, no endocrine disruption was observed, and the European Scientific Committee on Consumer Products has also concluded that 4-MBC poses no risk to humans [34, 35]. Therefore, whilst some transcriptional changes were observed in mice in this study, this does not necessarily translate to adverse effects in humans. No statistically significant changes in transcript levels were identified in mice treated with the ZnO sunscreen with or without UVR, or the TiO_2_ sunscreen with no UVR after 36 weeks of exposure.

The anatase form of TiO_2_, which is much more photocatalytic than the rutile phase, is incorporated into some commercially-available sunscreens, and is damaging to cultured human skin cells [36]. Coating or doping TiO_2_ nanoparticles prior to their incorporation into sunscreen is a method by which their photocatalytic activity can be substantially reduced. Nevertheless, it may be reassuring to note that the TiO_2_ nanoparticles in the sunscreen used here, which had photocatalytic properties similar to Degussa P25 ([37]; personal communication, [38]), did not produce adverse effects in the long-term when tested under the conditions of our study. Importantly, however, we did not irradiate unprotected skin following removal of the sunscreen. Future work might expose skin with residual TiO_2_ particles left in skin furrows and in hair follicles after washing. Once exposed to UVR rather than covered by recommended amounts of sunscreen formulation [39], the biological impact of photocatalytic particles in skin could be better ascertained.

No pathologies, apart from UVR-induced skin neoplasms in mice receiving UVR treatments but no sunscreen, were found to be due to a common cause. However, the relatively high incidence of adverse events in the ZnO + UVR group in Series 1, which resulted in five of the 10 mice in this treatment group failing to reach the experimental endpoint, required careful consideration. Mice in this group that did reach the experimental endpoint did not show treatment-related organ pathologies, or altered gene expression profiles. Testing for microbial infection was negative. The adverse events for three of the five mice that did not reach the experimental endpoint (two euthanized at week 20, and one at 32) were not histologically ascribed to a common cause, whilst the other two were found dead in their cage partially cannibalised (at weeks 10 and 13), and therefore no necroscopy was performed and a cause of death was not determined. While Zn excretion was not monitored throughout the experiment, it appears unlikely that the mice in the ZnO + UVR group were experiencing side effects linked to excess Zn given no long-term tissue accumulation was detected. Consistent with this, previous work has shown that mechanisms in rodents [28] and humans [40] are sufficient to maintain homeostasis, even under conditions of excess Zn. Furthermore, when we repeated the long-term exposure to ZnO sunscreen in Series 2, the high rate of adverse events in ZnO + UVR was not reproduced, and significant pathologies were not detected in the repeat group post mortem. We therefore conclude that the adverse events for ZnO + UVR in Series 1 were either unrelated to each other, were related to UVR-mediated immune suppression in the slightly higher UVR used in the Series 1 protocol, and/or were due to an as-yet unidentified factor in the cage.

## Conclusions

In conclusion, this study addressed a perceived knowledge gap regarding the safety of long-term use of sunscreens containing metal oxide nanoparticles. Sunscreens containing ZnO or TiO_2_ nanoparticles, organic UVR filters, or a mixture of both were applied topically once per week over 36 weeks to immune-competent hairless mice, a subset of whom were subsequently exposed to UVR (Series 1). The experiment with minor changes was repeated for the ZnO sunscreen (Series 2). Mice across both Series were assessed for histopathological changes compared to untreated mice, as well as for alterations in the concentrations of major organ tissue Zn (ZnO sunscreen-treated mice, Series 1), and liver tissue Ti (TiO_2_ sunscreen-treated mice, Series 1), and whole genome transcriptional profiling (all mice, Series 1). The major finding was that use of any of the sunscreens protected mice from developing a range of adverse UVR-mediated malignant and non-malignant skin neoplasms. Use of the ZnO sunscreen was not associated with statistically significant variations in serum and internal organ tissue Zn, whereas mice receiving applications of the TiO_2_ sunscreen showed a very small increase in liver Ti, possibly due to chronic low-level ingestion of residual TiO_2_ after washing. Neither of these sunscreens was associated with substantial alteration at the level of gene transcripts. In contrast, the transcriptional profile was substantially disrupted in mice receiving treatments of the organic sunscreen, which is of interest in light of work elsewhere linking 4-MBC with endocrine disruption in rodents (but not in humans). Overall, the results arising from the current study showed that long-term use of sunscreens containing ZnO or TiO_2_ nanoparticles provided protection against UVR-induced skin neoplasms and did not result in adverse biological outcomes in a hairless mouse model.

## Methods

### Series 1

#### Sunscreens used in this study

The three types of sunscreen (Table 5) used in this study, each with an SPF of 30+, were purchased from a local retail store in Sydney, Australia. One sunscreen (ZnO) contained only ZnO (200 mg/g) as its active ingredient. A second sunscreen (TiO_2_) contained a mixture of TiO_2_ (40 mg/mL) and organic chemicals (OCM, 70 mg/mL; B-MDM, 40 mg/mL) as UV-active ingredients. This particular sunscreen was selected on the basis of its similarity to a sunscreen that had elsewhere been shown to contain nanoparticles with photocatalytic properties similar to Degussa P25, a commercially-available product comprising both the anatase and rutile crystal phases of TiO_2_ nanoparticles ([37]; personal communication). The third sunscreen (organic) contained only organic chemicals as active ingredients (OCM, 99 mg/mL; B-MDM, 19.8 mg/mL; 4- 4-MBC, 39.6 mg/mL; octocrylene, 9.9 mg/mL), and was prepared by the same manufacturer as the TiO_2_ sunscreen. The TiO_2_ and organic sunscreens both contained OCM and B-MDM, albeit at different concentrations, whereas 4-MBC and octocrylene were present in the organic sunscreen only.

**Table 5:** Active ingredients of sunscreens used in this study

Sunscreen	Active ingredients
ZnO	ZnO (200 mg/g)
TiO_2_	TiO2 (40 mg/mL)
	OCM (70 mg/mL)
	B-MDM (40 mg/mL)
Organic	OCM (99 mg/mL)
	B-MBM (19.8 mg/mL)
	4-MBC (37.6 mg/mL)
	Octocrylene (9.9 mg/mL)

#### Nanoparticle characterisation

The presence of nanoparticles in the ZnO and TiO_2_ sunscreens was confirmed by extracting, imaging, and size-measuring the particles. Particles were extracted using a previously described method [37]: washing a 2-3 mg sample from each sunscreen sequentially twice through hexane (30 mL), twice through ethanol (30 mL), twice through water (30 mL), and once through acetone (30 mL) for approximately one minute each wash. The samples were centrifuged after each wash (room temp, 2 min, 7000 rpm), and supernatants were removed. It is possible that this extraction procedure may have altered either the particle size, agglomeration state, and/or surface characteristics compared to *in situ* sunscreen. Following the final acetone wash, the samples were air-dried, and the particles were analysed for their size and shape by transmission electron microscopy (TEM), using a Tecnai 12 TEM (FEI, Eindhoven, Netherlands) operating at 120 kV with a variety of magnifications. For imaging, the powders were dispersed in ethanol to form milky suspensions, and 4 μL aliquots were applied to 400-mesh carbon-coated grids freshly glow-discharged for 15 s in nitrogen. The samples were allowed to settle for approximately 1 min with excess blotted using filter paper. Images were recorded using a MegaView III CCD camera (Olympus) and sizes of nanoparticles determined using Image J software (NIH) calibrated via the embedded scale bar.

#### Animal housing conditions

Female, albino, immune-competent, hairless SKH:QS mice were supplied in-house by the CSIRO Animal House from an in-bred colony that had been developed from a seed cohort originally supplied by Sydney University, Australia [28]. Animals entered the study at 8 wks of age. Animal experiments were approved by the CSIRO North Ryde Animal Ethics Committee. For the duration of the treatment period, mice were housed in groups of 10 in open-topped polycarbonate cages in an isolated temperature- (~21 °C) and moisture- (55-65 % relative humidity) controlled room with a 14 h light/10 h dark cycle, and had *ad libitum* access to water and Gordon’s rat and mouse pellets (Gordon’s Speciality Stock Feeds, Australia).

#### Source of UVR

Mice were irradiated beneath a purpose-built scanning multi-lamp array, designed and constructed by the University of Sydney and the CSIRO Division of Applied Physics to simulate the solar spectrum. The irradiating source, held 30 cm above the irradiance table surface, comprised 8 X 140 W UVA lamps, 6 X 15 W UVB lamps, 25 X 250 W tungsten halogen lamps, and 8 X 140 W 5’actinic blue-light lamps. UVC wavelengths were excluded from the spectrum by filtering the emitted light through cellulose acetate film, and the absence of UVC wavelengths was confirmed spectroradiometrically. The UVB lamps were held within a scanning apparatus with a total scanning area of 140 cm x 50 cm, and scanning frequency between 3-12 scans over the scanning area per minute. All lamps were supplied by Commercial and Domestic Lamp Supplies, NSW Australia. Irradiance was measured and characterised by a portable, calibrated (NIST Calibration # 08042932) StellarNet UV-VIS-NIR spectroradiometer (EPP2000C UV-VIS CXR, Warsash Scientific, NSW Australia). Lamps were switched on 15 min prior to use to allow UV output to stabilise. Room temperature was stabilised by air-conditioning.

#### Treatment regime

For Series 1, eighty mice were divided into eight treatment groups of 10, and each mouse was treated once per wk. In comparison to Australian standards, this represents an infrequent application of sunscreen. Conversely, weekly exposure to sunscreen may exceed the application frequency in far northern hemisphere countries. Mice in Control-UVR and Control + UVR received no sunscreen; ZnO-UVR and ZnO + UVR received topical applications of the ZnO sunscreen; TiO_2_-UVR and TiO_2_ + UVR received topical applications of the TiO_2_ sunscreen; and Organic-UVR and Organic + UVR received topical applications of the organic sunscreen. Control-UVR, ZnO-UVR, TiO_2_-UVR and Organic-UVR received no UVR treatment, and Control + UVR, ZnO + UVR, TiO_2_ + UVR and Organic + UVR received approximately 29 kJ/m^2^ UVR (comprising an average of 27 kJ/m^2^ UVA and 2 kJ/m^2^ UVB) per treatment. This dose was selected on the basis of pilot data that had shown this dose to be sufficient to elicit a statistically-significant thickening of the dorsal skin area 48-72 h post-irradiation in the absence of any sun-protection factor (data not shown). All mice received a total of 30 treatments over the course of 36 wks. The last treatment was on week 36 followed by necroscopy approximately 24 h after the last irradiation.

Prior to each sunscreen application, and for the duration of each weekly treatment, mice in all groups were fitted with Elizabethan collars (Braintree Scientific, USA) to prevent self-grooming and consequent large-scale ingestion of the sunscreen formulations. A preliminary behavioural study conducted at the request of the CSIRO North Ryde Animal Ethics Committee showed that mice were not unduly bothered by the collars. Bedding was removed from cages and replaced with a lining of paper towel to minimise sunscreen removal by rubbing. Using a gloved fingertip, sunscreen was applied at 2 mg/cm^2^ [39] to the head, ears, back, sides and tail of each mouse, and sunscreens were left to equilibrate for 20 min before UVR exposure, according to manufacturers’ instructions. Although only dorsal skin thickness was measured, sunscreen was applied to all skin areas exposed to UVR to prevent localised sunburn. The total volume of sunscreen applied varied slightly per mouse according to its size, but on average approximated 110 μL.

For UVR treatments, mice in the same treatment group were placed unrestrained in an open-topped cage placed within a larger bed of ice that kept the cage cool under the irradiating lamps. To avoid differences caused by “hot” and “cold” irradiation spots within the total irradiation area of 140 cm x 50 cm, cages were placed in the same location on the irradiation table one at a time. Time under lamps to deliver the standard UVR dose varied slightly with each treatment and was calculated just prior to each individual irradiation using SpectraWiz® software packaged with the spectrometer. Thus, we attempted to standardise the UVR dose received by different treatment groups. Following irradiation, mice were returned to their cages lined with fresh paper towel for 2 h, after which collars were removed and sunscreens were washed from the mice using luke-warm pH balanced soapy water, and soft cotton pads. Mice were then dried using fresh cotton pads, and returned to cages with clean standard bedding until their next weekly treatment. Mice receiving no applications of sunscreen were sham treated in that they had Elizabethan collars applied, were held in cages lined with fresh paper towel for 2 h, after which they were washed. Mice were not observed to groom each other or themselves during treatment periods. Once per month, the thickness of the dorsal skin on each mouse was measured 72 h after the last irradiation using electronic callipers (Mitutoyo Absolute Digimatic CD-6”C).

After 36 wks (30 treatments), under anaesthesia induced using an intraperitoneal injection of Xylazine [(50 mg/kg)/ketamine (50 mg/kg)], mice were weighed and then euthanized by cervical dislocation, and skin neoplasms were counted and classified macroscopically. The dorsal skin was carefully dissected, mounted on cardboard, noting the head-tail orientation, and stored on ice in 1 X Histochoice tissue fixative (Astral Scientific, Australia) for histopathology. Major internal organs (brain, liver, spleen, kidneys, lung and heart) were retrieved, weighed and sectioned. Sections were either stored in 4 % neutral buffered formalin at room temperature for histopathology, or snap frozen in liquid nitrogen and stored at -80 °C for other analyses.

#### Histopathology

Skin samples were processed within 24 h of dissection, and sectioned by a commercial provider (VPS, University of Sydney, Australia). All other organs were processed, sectioned and stained by a commercial provider (Gribbles Veterinary Pathology, Australia). Histopathological analysis of all samples was performed by the same veterinary pathologist on a commercial basis (Gribbles Veterinary Pathology, Australia). The histopathologist was not privy to the nature of each treatment with the exception of Control-UVR, which was identified as the untreated control group to enable statistical comparisons. Abnormalities identified in organs were classified, typically on their degree of severity, and the incidence within a treatment group was then compared to the incidence across all groups.

#### Measurement of tissue Zn by ICP-MS

Total Zn concentrations were measured by ICP-MS in skin, brain, liver, spleen, kidney and lung tissues harvested from Control-UVR, Control + UVR, ZnO-UVR and ZnO + UVR. Frozen samples (0.04 g wet mass) were processed and analysed by a commercial provider (Advanced Analytical Australia). Five samples were measured for each treatment group except for Control + UVR skin, for which four representative samples were measured.

#### Measurement of liver Ti by ICP-MS

Total Ti concentrations in livers harvested from mice in Control-UVR, TiO_2_-UVR and TiO_2_ + UVR were determined in-house by ICP-MS. Liver tissue (0.01 - 0.05 g wet mass) was weighed into 50 ml PFA beakers with 10 ml double-distilled concentrated nitric acid (Savillex DST-100) and 0.5 ml hydrogen peroxide (≥30 %, TraceSELECT Ultra, Fluka). The samples were open-vessel digested for 1 h at 70 °C, and further closed-vessel digested (lids added) at 70 °C for 2 h. Vessel lids were removed and solutions evaporated to dryness at 70 °C. The samples were further digested in 5 ml of double-distilled concentrated nitric acid and 0.5 ml hydrofluoric acid (48 %) at 70 °C for 2 h. Following digestion, samples were evaporated to dryness, resuspended in 2 % nitric acid, and stored in PFA vessels until analysis. Total Ti concentrations in solutions were determined using ICP-MS (Agilent 7700) by monitoring mass/charge (m z^-1^) 49. Collision cell mode was used for ICP-MS analysis using helium as a collision gas at a flow rate of 4 ml min^-1^. The ICP-MS instrument was optimised daily to minimise double charged and oxide interferences to < 2 % (m z^-1^ 70/ m z^-1^ 140) and < 1 % (m z^-1^ 156/ m z^-1^ 140), respectively. Four to six representative samples were measured for each treatment group. Selected samples were spiked with 10 μg/L solution of Ti to examine the accuracy of the digestion and analysis procedures. Mean spike recoveries were in good agreement with the tissue spiked Ti concentrations (95 ± 2.7 %, *n* = 6).

#### Statistical analyses of data

All statistical analyses of data were performed using GraphPad Prism 6.00 (GraphPad Software, USA), unless otherwise specified. Histological findings in treatment groups were assessed for statistical significance compared to Control-UVR using a two-sided Fisher’s exact test (alpha < 0.05). Differences between groups for skin thickness and/or organ concentrations of Zn and Ti were assessed for statistical significance by one-way ANOVA with Tukey’s Multiple Comparisons Test (if three or more groups), or unpaired t-tests with Welch’s Correction (if only two groups), with significance set at *p* < 0.05.

#### Analysis of 28,853 gene transcripts in mouse liver by whole-genome expression profiling

Liver tissue was chosen for an analysis of altered gene expression in treated mice relative to controls on the basis of previous work indicating a high exchange in the liver of Zn from topically applied sunscreen with endogenous Zn, as well as the development of an inflammatory response from short-term exposure to a sunscreen formulation without nanoparticles [28].

Procedures for RNA isolation from mouse liver samples (30 mg wet weight) and measurements of concentration and quality assurance were performed as described elsewhere [28] using a NucleoSpin® RNA II kit (Macherey-Nagel, Scientifix), a NanoDrop DN-1000 spectrophotometer (Biolab), and an Agilent 2100 Bioanalyzer (RNA 6000 Nanochip™, Agilent Technologies), respectively. Samples were prepared for microarray analysis using the Affymetrix GeneChip Mouse Gene 1.0 ST Array Combo kit and Hybridisation, Wash and Stain Kit (Millennium Science) following manufacturer’s instructions. Microchips were hybridised (17 h, 45 °C, 60 rpm in an Affymetrix 640 GeneChip® Hybridization Oven), then washed using an Affymetrix GeneChip® Fluidics Station 450, and scanned using an Affymetrix 7G GeneChip® Scanner. All microchips passed the associated quality-control procedures recommended by Affymetrix. Representative samples from six different mice in each treatment group were prepared.

The microchips were processed in six batches of eight chips by the same operator, following the same procedure, with each batch processed on a different day. Each batch contained one microchip from each of the eight groups to minimize the consequences of batch effects on downstream analysis [41]. Transcripts were calculated as being up- or down-regulated compared to untreated mice as described elsewhere [41] using Matlab Bioinformatics and Statistical toolboxes, and normalized using robust multi-array average. Batch effects were removed, and control for false discovery was set at 10 % using in-house tools [42]. Transcripts identified as being differentially expressed with statistical significance compared to Control-UVR were analyzed using Ingenuity Pathways Analysis (IPA) (Ingenuity® System, www.ingenuity.com), taking into account whether they were under-expressed or over-expressed.

### Series 2

To further investigate adverse events resulting in the unscheduled deaths of five of 10 mice in the ZnO + UVR group of Series 1, the experiment was repeated as described above for the control and ZnO ± UVR treatment groups with minor modifications. To distinguish a potential treatment effect from an unidentified cage-associated factor, the 10 mice in each treatment group were housed in two cages each of five mice rather than one cage of 10 as in Series 1. The weekly UVR dose of 29 kJ/m^2^ applied in Series 1 had been selected on the basis that it elicited a statistically significant level of skin thickening in pilot experiments; however exposure to UVR has been associated in mice and humans with immune suppression [43], and therefore the total UVR dose was lowered slightly in Series 2 to 27 kJ/m^2^, to bring it closer to doses used elsewhere [17, 18, 44, 45]. Furthermore, whilst bearing in mind that extended exposure to the UVA component of UVR is hardly benign [19, 20], UVB can efficiently induce direct damage to cellular components including DNA and proteins [3], and increasing UVA levels relative to UVB has been associated with a decrease in UVB-mediated cell apoptosis [21]. Therefore, the UVB dose used in Series 2 was lowered from 2 (used in Series 1) to 1.4 kJ/m^2^ and the UVA dose was adjusted from 27 to 25.6 kJ/m^2^, resulting in a UVA:UVB ratio greater than 17 as found under meteorological conditions [20], and more closely reflecting the relative percentages UVA and UVB in total UVR on a summer’s day at noon in Northern European latitudes [22]. It is important to emphasize, however, that both Series 1 and 2 were not designed to be UVR carcinogenesis experiments, but rather experiments to test the long-term biological impact of using sunscreens, with UVR as a variable. Mice in Series 2 received 32 treatments over 36 wks, using the same ZnO sunscreen as for Series 1. The last treatment was on week 36 followed by necropsy approximately 24 h after the last irradiation. Post-mortem, histological analyses were performed by the same veterinary histopathologist as in Series 1. Tissue Zn concentrations were not measured in Series 2, and global gene-expression profiling in liver was not performed.

## Abbreviations

μg, microgram; μL, microliter; °C, degrees celsius; 4-MBC, 4-methylbenzylidene camphor; AE, adverse event; BAC, bronchioalveolar carcinoma; B-MDM, butyl methoxydibenzoylmethane; CFS, cutaneous fibrosarcoma; cm, centimeter; COLIPA, now Cosmetics Europe; DNA, deoxyribonucleic acid; E, endometritis; EU, European Union; g, gram; h, hour; HC, Haemolytic crisis; ICP-MS, inductively coupled plasma mass spectrometry; kg, kilogram; kj, kilojoule; LL, lymphocytic lymphoma; m, meter; mg, milligram; min, minute; mL, milliliter; mm, millimeter; N, nephropathy; n, number; OCM, octylmethoxycinnamate; OS, osteosarcoma; P, peritonitis; PA, pulmonary adenoma; rpm, revolutions per minute; SCC, squamous cell carcinoma; SCP, squamous cell papilloma; SEM, standard error of the mean; TEM, transmission electron microscopy; TGA, Therapeutic Goods Administration; Ti, titanium; TiO_2_, titanium dioxide; UV, ultraviolet; UVA, ultraviolet A; UVB, ultraviolet B; UVC, ultraviolet C; UVR, ultraviolet radiation; W, watts; wk(s), week(s); Zn, zinc; ZnO, zinc oxide
